# Tepotinib Inhibits Several Drug Efflux Transporters and Biotransformation Enzymes: The Role in Drug-Drug Interactions and Targeting Cytostatic Resistance In Vitro and Ex Vivo

**DOI:** 10.3390/ijms222111936

**Published:** 2021-11-03

**Authors:** Dimitrios Vagiannis, Youssif Budagaga, Anselm Morell, Yu Zhang, Eva Novotná, Adam Skarka, Sarah Kammerer, Jan-Heiner Küpper, Ivo Hanke, Tomáš Rozkoš, Jakub Hofman

**Affiliations:** 1 Department of Pharmacology and Toxicology, Faculty of Pharmacy in Hradec Králové, Charles University, Heyrovského 1203, 500 05 Hradec Králové, Czech Republic; dimitris_vagiannis@yahoo.gr (D.V.); budagagy@faf.cuni.cz (Y.B.); zhangyu2@faf.cuni.cz (Y.Z.); 2 Department of Biochemical Sciences, Faculty of Pharmacy in Hradec Králové, Charles University, Heyrovského 1203, 500 05 Hradec Králové, Czech Republic; morellga@faf.cuni.cz (A.M.); novotne7@faf.cuni.cz (E.N.); 3 Department of Chemistry, Faculty of Science, University of Hradec Králové, Hradecká 1285, 500 03 Hradec Králové, Czech Republic; adam.skarka@uhk.cz; 4 Institute of Biotechnology, Brandenburg University of Technology Cottbus-Senftenberg, Universitätsplatz 1, 01968 Senftenberg, Germany; sarah.kammerer@b-tu.de (S.K.); jan-heiner.kuepper@b-tu.de (J.-H.K.); 5 Department of Cardiac Surgery, Faculty of Medicine, Charles University and University Hospital Hradec Králové, Sokolská 581, 500 05 Hradec Králové, Czech Republic; ivo.hanke@fnhk.cz; 6 The Fingerland Department of Pathology, Faculty of Medicine, Charles University and University Hospital Hradec Králové, Sokolská 581, 500 05 Hradec Králové, Czech Republic; tomas.rozkos@fnhk.cz

**Keywords:** tepotinib, non-small cell lung cancer, multidrug resistance, drug interaction, ABC transporter, cytochrome P450

## Abstract

Tepotinib is a novel tyrosine kinase inhibitor recently approved for the treatment of non-small cell lung cancer (NSCLC). In this study, we evaluated the tepotinib’s potential to perpetrate pharmacokinetic drug interactions and modulate multidrug resistance (MDR). Accumulation studies showed that tepotinib potently inhibits ABCB1 and ABCG2 efflux transporters, which was confirmed by molecular docking. In addition, tepotinib inhibited several recombinant cytochrome P450 (CYP) isoforms with varying potency. In subsequent drug combination experiments, tepotinib synergistically reversed daunorubicin and mitoxantrone resistance in cells with ABCB1 and ABCG2 overexpression, respectively. Remarkably, MDR-modulatory properties were confirmed in ex vivo explants derived from NSCLC patients. Furthermore, we demonstrated that anticancer effect of tepotinib is not influenced by the presence of ABC transporters associated with MDR, although monolayer transport assays designated it as ABCB1 substrate. Finally, tested drug was observed to have negligible effect on the expression of clinically relevant drug efflux transporters and CYP enzymes. In conclusion, our findings provide complex overview on the tepotinib’s drug interaction profile and suggest a promising novel therapeutic strategy for future clinical investigations.

## 1. Introduction

For decades, lung cancer has represented the deadliest type of malignancy within oncological diseases. The clinical outcomes of its treatment are hampered by the development of drug resistance, which can be based on pharmacodynamic and pharmacokinetic principles [1]. ATP-binding cassette (ABC) drug efflux transporters together with cytochrome P450 (CYP) biotransformation enzymes play crucial pharmacokinetic roles through orchestrating absorption, distribution and elimination of numerous drugs. Due to this feature, some members of these superfamilies provoke clinically relevant drug interactions (DIs) [2,3]. At the same time, ABCB1, ABCG2, ABCC1 and CYP3A4 were recognized as important pharmacokinetic mechanisms of oncological multidrug resistance (MDR). These proteins are overexpressed in several tumor types, where they decrease the efficacy of anticancer drugs by efflux or metabolism, respectively [4,5].

Tepotinib (trade name Tepmetko) is a novel c-MET tyrosine kinase inhibitor that was recently approved in Japan for the treatment of patients with advanced non-small cell lung cancer (NSCLC) harboring a *MET* exon 14 skipping mutation [6]. In February 2021, US Food and Drug Administration (FDA) approved the drug for the treatment of adult patients with metastatic NSCLC [7]. In the present study, we aimed to investigate the possible potential of tepotinib to mediate pharmacokinetic drug interactions. In addition, we evaluated whether these interactions might be utilized for combating MDR both in vitro and in vitro.

## 2. Results

### 2.1. Tepotinib Potently Inhibits ABCB1 and ABCG2 Transporters

**Figure 1:**
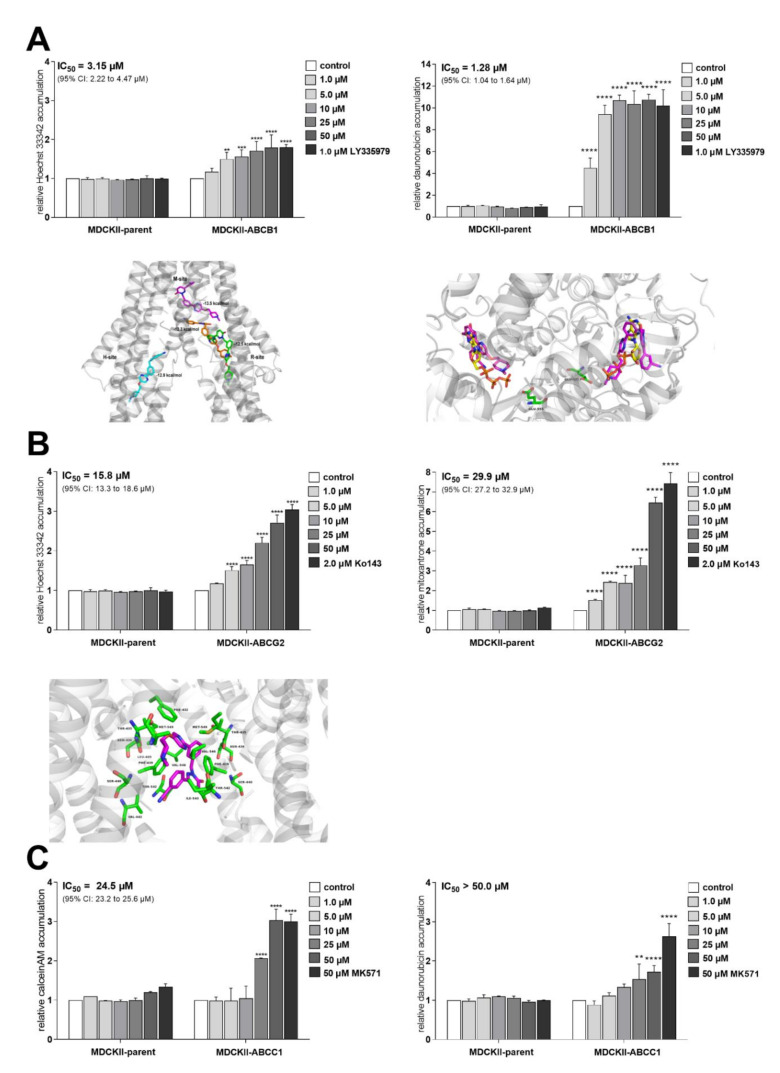
Effect of tepotinib on the activity of MDR-associated ABC transporters in vitro and in silico. Graphs from accumulation assays in ((**A**) **up**) MDCKII-ABCB1, ((**B**) **up**) MDCKII-ABCG2 and (**C**) MDCKII-ABCC1 cells are shown along with the results from ((**A**) **bottom**) ABCB1 and ((**B**) **bottom**) ABCG2 docking. Tepotinib is depicted as ((**A**) **bottom left**) colored sticks or ((**A**) **bottom right** and (**B**) **bottom left**) magenta sticks; ((**A**) **bottom right**) ATP molecules that originally co-crystallized with the protein backbone are shown as yellow sticks; ((**A**) **bottom right** and (**B**) **bottom left**) interacting amino acid residues as green sticks and are labeled.

In accumulation studies, tepotinib potently inhibited the ABCB1 transporter yielding low micromolar IC_50_s for both model substrates (Figure 1A). ABCG2 was also significantly inhibited at the majority of concentration points, although with lower potency (Figure 1B). Finally, a negligible extent of interaction was observed for the ABCC1 transporter (Figure 1C). The experimental results were confirmed by molecular modeling. In the case of ABCB1, tepotinib showed the highest affinity for the M- and H-sites (Figure 1A bottom left). Furthermore, the docking suggested tepotinib interactions with residues involved in the binding of ATP (Figure 1A bottom right). However, only crystal structure may prove the affinity of tepotinib for nucleotide binding domains, because an unfavorable interaction with Lys-1076 has been predicted (Supplementary Materials Figure S2). For ABCG2, our results show that tepotinib may stabilize the inward-facing conformation of this transporter and prevent binding of ATP (Figure 1B bottom).

### 2.2. Tepotinib Inhibits Several Recombinant CYP Isoforms, but Not CYP3A4 Enzyme in Intact Cells

**Figure 2:**
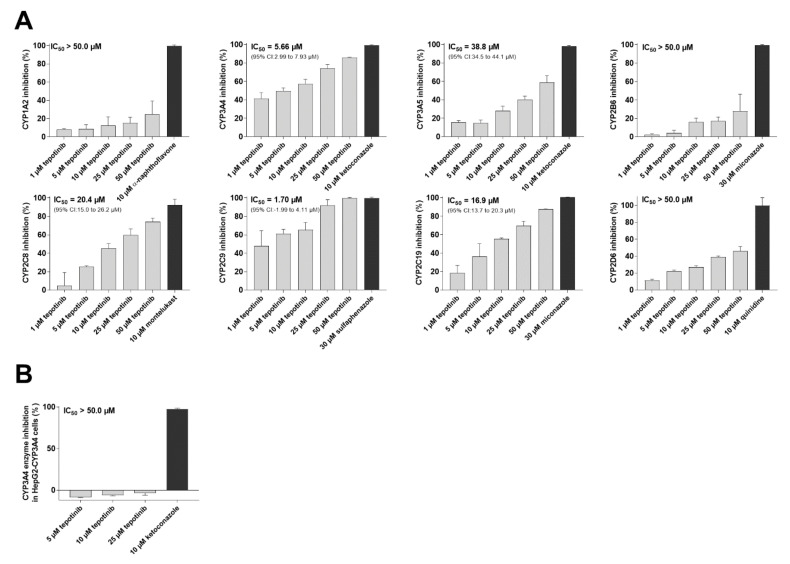
Effect of tepotinib on the activity of clinically relevant human CYP isoforms. (**A**) Screening of inhibition of recombinant enzymes by using Vivid CYP450 Screening Kits. Raw fluorescence data were normalized against control 0% and 100% activity values. Maximal (100%) control value was obtained from reaction that contained only the enzyme and 0.5% DMSO with no drug. Control 0% activity value was represented by sample consisting of 0.5% DMSO and the enzyme solvent buffer without the enzyme. 0.5% DMSO was present in all dilutions of tepotinib/model inhibitors to avoid results’ distortion by fluctuation in DMSO levels. (**B**) Effect on CYP3A4 metabolic activity in HepG2-CYP3A4 cells. Metabolic values were first normalized to viability data. Resulting values were then re-normalized as % of inhibition. Control value representing 100% activity was obtained from cells exposed to vehicle-containing media and IPA substrate, while 0% activity control came from cell-free wells, into which vehicle-containing media and IPA substrate were added.

Using Vivid CYP450 Screening Kits, tepotinib was found to be a potent inhibitor of CYP2C9 (IC_50_ = 1.70 µM) and a moderate inhibitor of CYP3A4 (IC_50_ = 5.66 µM). Lower level of interaction was observed in the case of CYP2C8 (IC_50_ = 20.4 µM) and CYP2C19 (IC_50_ = 16.9 µM), and CYP1A2, CYP2B6, CYP2D6 and CYP3A5 were inhibited negligibly by tepotinib (Figure 2A). Recently, CYP3A4 was confirmed to be a mediator of docetaxel resistance [5]. Therefore, we used HepG2-CYP3A4 cellular assay to verify, whether tepotinib might have a potential to act as a modulator of this kind of resistance. In contrast to data from the recombinant enzyme, no inhibition of CYP3A4 enzyme was monitored in intact HepG2-CYP3A4 cells (Figure 2B). This inconsistency can arise from several factors such as limited membrane penetration, efflux or impaired influx by drug transporters, and trapping in endosomes, among others [8]. Taken together, tepotinib failed to exhibit the potential for overcoming CYP3A4-mediated docetaxel resistance [9].

### 2.3. Tepotinib Effectively Modulates ABCB1- and ABCG2-Mediated Cytostatic Resistance In Vitro

In the follow-up studies, we evaluated MDR-reversal properties of tepotinib in in vitro cellular models with ABCB1 or ABCG2 overexpression. Tepotinib concentrations (5 or 10 µM for ABCB1 or ABCG2, respectively), which were negligibly cytotoxic in model cell lines and showed significant transporter inhibition, were tested in drug combinations.

**Figure 3:**
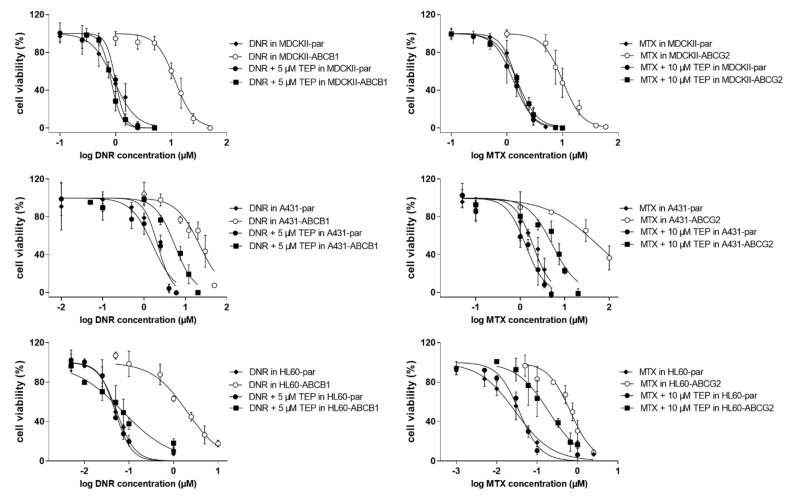
Tepotinib reverses daunorubicin and mitoxantrone resistance in vitro. Drug combinations were conducted using MTT/XTT proliferation test. Absorbance values from cells treated with vehicle-containing media and 40% DMSO in media were used as 100% and 0% viability controls for data normalization, respectively. DNR, daunorubicin; MTX, mitoxantrone; TEP, tepotinib.

**Figure 4:**
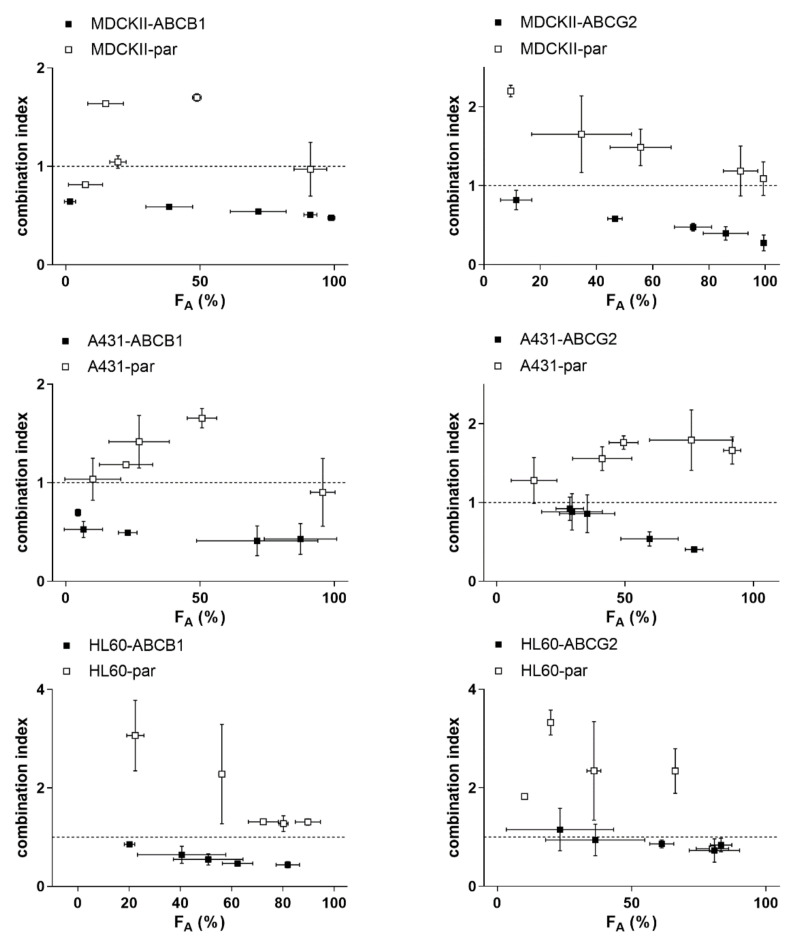
Combination index (CI) analysis of the drug combinations shown in Figure 3. Combination outcomes can be synergistic (CI < 0.9), additive (0.9 < CI < 1.1), or antagonistic (CI > 1.1). F_A_, the fraction of cells affected.

**Figure 5:**
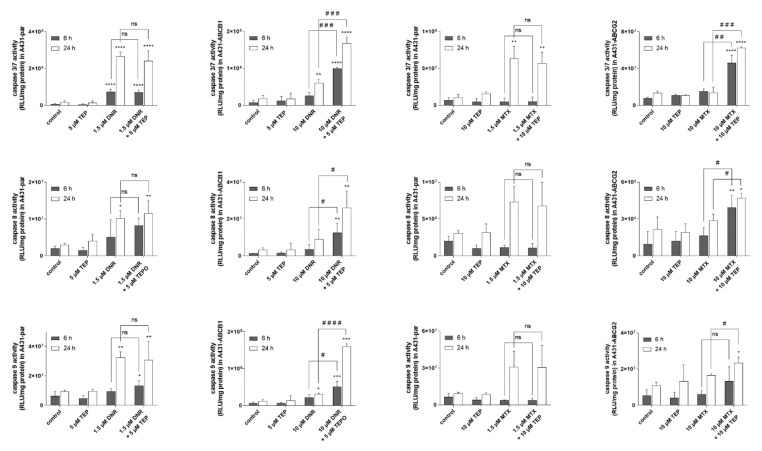
Caspases’ activities assessments. * one-way *ANOVA* followed by Dunnett’s post hoc test (comparison of all treated variants to control), ^#^ two-tailed unpaired *t*-test (single drug vs. combined treatments). DNR, daunorubicin; F_A_, fraction of cells affected; MTX, mitoxantrone; TEP, tepotinib.

Tepotinib significantly sensitized transporter-overexpressing MDCKII, A431 and HL60 cells to daunorubicin and mitoxantrone, while no such effects were observed in respective parent cell lines (see IC_50_ shifts in Figure 3 and associated analysis in Supplementary Materials Table S2). Combination index analysis showed a substantial difference between combination outcomes; synergism and antagonistic/additive effects were predominantly recorded in transporter-expressing and parental cell lines, respectively (Figure 4). These data clearly confirm that tepotinib-mediated inhibition of ABCB1 and ABCG2 plays an essential role in MDR reversal effect. In addition, results of caspases’ activities assessments demonstrated that efficient activation of apoptosis is the molecular mechanism hidden beyond the synergistic effect of drug combinations in transporter-expressing cells (Figure 5).

### 2.4. Tepotinib Overcomes MDR in Ex Vivo NSCLC Explants

**Figure 6:**
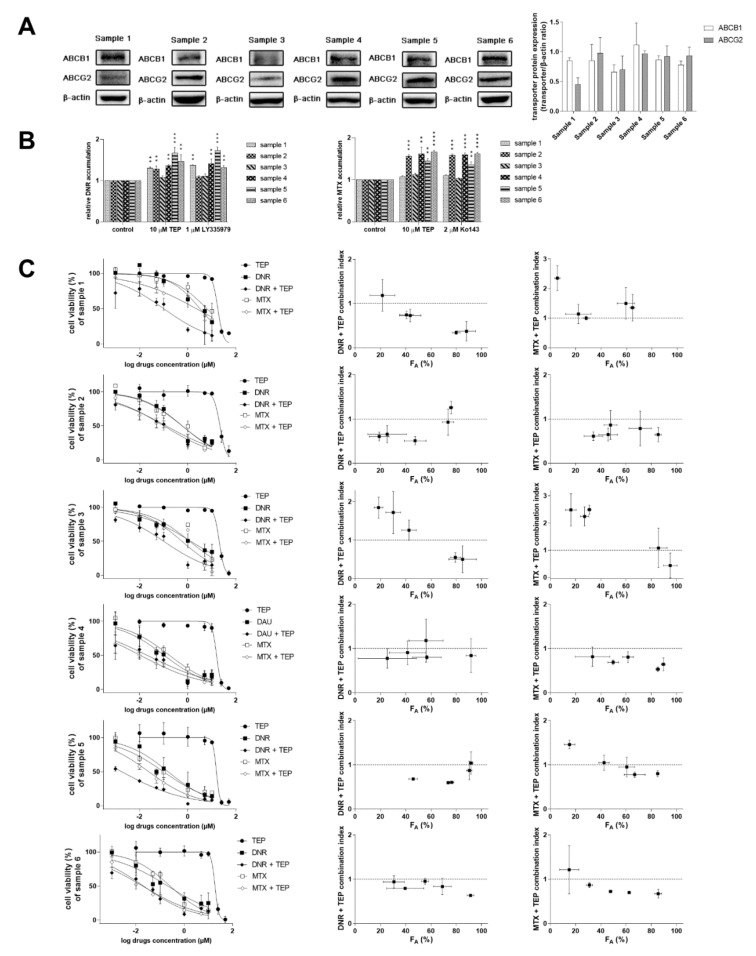
Tepotinib synergizes with MDR victim cytostatics in patient-derived NSCLC explants. (**A**) Expression levels of ABCB1 and ABCG2 (**left**, representative images; **right**, quantitative densitometric analysis). (**B**) Effect of tepotinib and model inhibitors on the accumulation of mitoxantrone and daunorubicin. (**C**) Antiproliferative effects of tepotinib, mitoxantrone, daunorubicin and their combination (10 µM tepotinib was applied as MDR modulator in combinations). Treatments with vehicle-containing media and 40% DMSO in media were used as 100% and 0% viability controls for data normalization, respectively. Combination index analysis of obtained data is shown near to viability curves. Combination outcomes can be synergistic (CI < 0.9), additive (0.9 < CI < 1.1), or antagonistic (CI > 1.1). DNR, daunorubicin; F_A_, fraction of cells affected; MTX, mitoxantrone; TEP, tepotinib.

The possible clinical impact of our in vitro drug combination results was evaluated in patient-derived NSCLC explants. We have selected six primary cultures with variable expressions of ABCB1 and ABCG2 (Figure 6A). Model inhibitors and tepotinib significantly increased the accumulation of model anticancer drugs in some of the primary samples, predominantly those with higher transporters’ expression levels (Figure 6B). Importantly, we also found a possible association between the outcomes of accumulation experiments and drug combination assays. Drug combinations in samples, which exhibited high levels of transporter expression and significant changes in the accumulation of probe anticancer drugs, predominantly generated synergistic outcomes. In contrast, major effects in samples with opposite properties were additivity or antagonism (Figure 6C). Mutual dependencies of outcomes of expression/accumulation/combination experiments were not absolute, likely due to primary nature of tumor samples (possible presence of SNPs in transporters’ sequence, varying expression of other transporters affecting accumulation of probe cytotoxic drugs, alterations in pharmacodynamic target etc.). However, these data confirm the potential of tepotinib to become a valuable MDR modulator for NSCLC patients with tumors positive for high ABCB1/ABCG2 expression.

### 2.5. Tepotinib Is a Substrate of ABCB1, but Not a Victim of ABCB1-Mediated MDR

**Figure 7:**
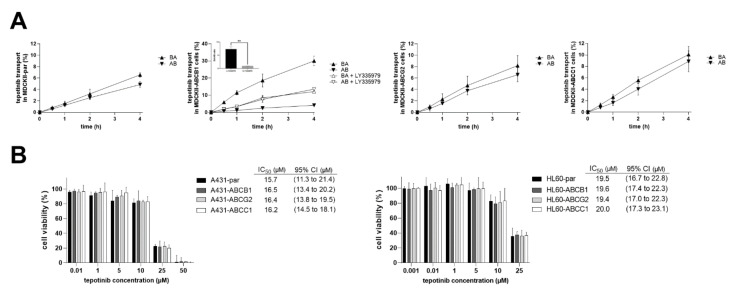
(**A**) Monolayer transport studies for 1 µM tepotinib in MDCKII cells. BA/AB ratio was calculated by dividing the percentage of tepotinib transported in basal-to-apical (BA) direction by that in apical-to-basal (AB) direction 4 h after drug’s addition. 100% control transport value was represented by the 1 µM solution of tepotinib from the same dilution, which was used for all tested variants. (**B**) Comparative viability studies in A431 and HL60 cells. Treatments with vehicle-containing media and 40% DMSO in media were used as 100% and 0% viability controls for data normalization, respectively. IC_50_ values from transporter-expressing cells were compared with those from parent cells, but no statistically significant differences were observed for any variant.

Transport studies designated tepotinib as ABCB1 substrate since the asymmetry of its transport in MDCKII-ABCB1 was aborted by LY335979, a specific ABCB1 inhibitor. No substrate affinities were observed in the case of ABCG2 or ABCC1 (Figure 7A). In contrast to expectation, we failed to detect any effect of ABCB1 presence on tepotinib’s efficacy in follow-up comparative proliferation experiments (Figure 7B).

### 2.6. Tepotinib Does Not Affect Gene Expression of ABC Transporters and CYP Enzymes

**Figure 8:**
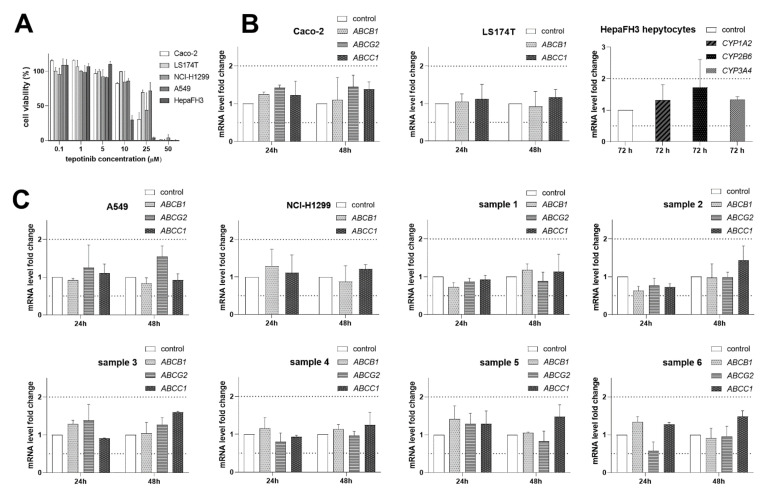
Gene induction studies with tepotinib. Treatments with vehicle-containing media and 40% DMSO in media were used as 100% and 0% viability controls for data normalization, respectively. (**A**) Effect of tested drug on the viability of examined cell lines. (**B**) qRT-PCR analysis of expression of ABC transporters and CYPs following exposure to 1.5 μM tepotinib in DIs-related models. (**C**) qRT-PCR analysis of expression of ABC transporters following exposure to 1.5 μM tepotinib in NSCLC cell lines and ex vivo NSCLC primary cultures. Dotted lines define the boundaries of downregulation/upregulation positivity based on the European Medicines Agency (EMA) DIs guidelines [10].

In the final study, tepotinib did not influence expression of clinically relevant ABC transporters and CYPs in DI-related models (Figure 8B) or NSCLC cell lines and primary NSCLC cultures (Figure 8C). Our findings suggest that tepotinib is not likely to act as a perpetrator of induction-based DIs or to influence MDR behavior of its target cells, respectively. The drug concentration was selected based on viability results in tested cells (Figure 8A) and maximum plasma concentration (C_max_) of a drug.

## 3. Discussion

Tepotinib (Tepmetko) is a novel anti-NSCLC agent recently approved in Japan and USA [6,7]. In this study, we explored pharmacokinetic interactions of tepotinib with ABC transporters and CYP drug metabolizing enzymes and investigated their possible exploitation for combating MDR in vitro and in vitro.

First, we described inhibition of several ABC drug efflux transporters and CYP isozymes by tepotinib. However, considering tepotinib’s steady state C_max_ observed at recommended dosing of 500 mg daily (2.62 µM) [11], unbound fraction (up to 3.25% of a dose) [12] and FDA/EMA guidelines [10,13], only ABCB1 and CYP2C9 inhibitions can be considered potentially clinically relevant for perpetrating systemic pharmacokinetic DIs. This statement is in accordance with the results of DI study in healthy subjects, where tepotinib significantly increased AUC and C_max_ of DI-sensitive ABCB1 substrate dabigatran etexilate, but it failed to exert the effect on CYP3A4 substrate midazolam [14].

Apart from systemic DIs, we focused on possible utilization of observed interactions to overcome MDR at intratumoral level. In drug combination studies, we proved the ability of tepotinib to modulate ABCB1- and ABCG2-mediated MDR to daunorubicin and mitoxantrone, respectively, in several in vitro models. Recently, tepotinib was demonstrated to potentiate the anticancer effect of paclitaxel and vincristine in in vitro KB drug resistant cell lines [15], which correlates with our findings. In addition to simple demonstration of the tepotinib’s ability to reverse MDR, we also focused on precise quantification of combination effects. We found synergistic outcomes in transporter-overexpressing models, which is an essential feature for possible practical application of our results. Synergistic combinations allow for dosage reduction and are thus widely used in clinical practice to increase the safety and/or efficacy of cancer treatment [16]. Next to in vitro models, we demonstrated that tepotinib modulates MDR in patient-derived NSCLC explants, confirming its clinical chemosensitizing potential. Importantly, following principles of personalized medicine would be strictly needed to find a patient population, which would benefit from the suggested therapeutic approach [4]. Unfortunately, no clues (such as description of intratumoral levels of tepotinib and the kinetics of its tumor uptake/excretion processes) are currently available to assist with reliable in vitro/ex vivo-in vivo extrapolation. Thus, clinical investigations will be necessary to verify real therapeutical value of our findings.

Although we characterized tepotinib as potent MDR modulator, this drug might be susceptible to resistance as well. While ABCG2 and ABCC1 can be clearly excluded as possible mediators of resistance to tepotinib, experiments on ABCB1 resulted in conflicting data. The drug was designated as ABCB1 substrate in monolayer transport assays, but functional presence of the transporter had no significant influence on its antiproliferative capacity in A431 and HL60 cellular models. This discrepancy can be explained by the relatively high lipophilicity of tepotinib with logP ≈ 3.64 (according to Advanced Chemistry Development Software version 11.02). It is well known that moderate transporter-mediated efflux of lipophilic substrates can be largely compensated for by passive diffusion [17]. Thus, standard MDR-victims (e.g., daunorubicin) are hydrophilic agents. However, it can take significant time to reach the concentration equilibrium even for lipophilic drugs. Our transport assays were 12-fold shorter than comparative proliferation experiments, which might lead to the manifestation of passive diffusion process selectively in proliferation experiments. According to the results of accumulation assays and molecular docking calculations, tepotinib could inhibit its own transport at higher concentrations, which could represent another mechanism resulting in contradictory outcomes of proliferation vs. transport studies. However, anticancer properties of tepotinib were not negatively affected by the presence of ABC transporters. Similar phenomenon, which makes tested drug an ideal MDR modulator, was observed in our previous study with ensartinib [9].

Finally, the pharmacological fate of tepotinib or co-administered drugs could be affected by induction of ABC transporter or CYP enzymes. To address this issue, we performed gene expression studies detecting *ABCB1*, *ABCG2*, *ABCC1*, *CYP1A2*, *CYP3A4* and *CYP2B6* in systemic and NSCLC cellular models following exposure to tepotinib. With respect to our results and EMA guidelines [10], we can anticipate that tepotinib does not exhibit potential for perpetrating systemic induction-based DIs on ABC transporters or CYP enzymes. In addition, it is not likely that the drug might induce the development of acquired pharmacokinetic MDR, which strengthens its potential value as MDR-combating agent. Lack of tepotinib’s induction effect on ABCB1 has recently been demonstrated [15]. However, other information about its effect on the expression of CYP enzymes and the rest of ABC transporters expression have not been given yet.

## 4. Materials and Methods

### 4.1. Chemicals and Reagents

Tepotinib was obtained from MedChem Express (New Jersey, NJ, USA). Hoechst 33342, daunorubicin, mitoxantrone, 3-(4,5-dimethyl-2-thiazolyl)-2,5-diphenyl-2H-tetrazolium bromide (MTT), 2,3-bis(2-methoxy-4-nitro-5-sulfophenyl)-5-[(phenylamino)-carbonyl]-2H-tetrazolium inner salt (XTT), dimethyl sulfoxide (DMSO), fetal bovine serum (FBS), phosphate buffered saline (PBS), fluorescein isothiocyanate-labeled dextran, phenazine methosulfate, CYP inhibitors (α-naphthoflavone, miconazole, montelukast, sulfaphenazole, quinidine and ketoconazole), hormones, penicillin/streptomycin, pituitary extract, triiodothyronine, phosphoethanolimine, ethanolamine, growth factors, gentamicin, collagenase, bovine serum albumin, trypsin inhibitor, protease inhibitor cocktail, Ficoll Paque Plus and cell culture media were purchased from Sigma Aldrich (St. Louis, MO, USA). Calcein AM, Vivid CYP450 Screening Kits, Pierce BCA protein Assay Kits, Pierce™ Coomassie Plus (Bradford, UK) Assay Reagent (cat. no. 23238), as well as DNase I, Reaction Buffer with MgCl2, 50 mM EDTA, oligo (dT)18 primers, 10 mM dNTPs for DNase digestion, RevertAid reverse transcriptase for cDNA synthesis, and Maxima Probe qPCR Master Mix for the analysis of *CYP1A2, CYP2B6* and *CYP3A4* expression in HepaFH3 cells was obtained from Thermo Fisher Scientific (Waltham, MA, USA). The InnuPREP RNA Mini Kit was bought from Analytik Jena (Jena, Germany). EvaGreen was obtained from Biotium (Fremont, CA, USA). LY335979 (zosuquidar) was obtained from Toronto Research Chemicals (North York, ON, Canada). Ko143 and MK571 were from Enzo Life Sciences (Farmingdale, NY, USA). Opti-MEM, Minimum Essential Medium (MEM) and media for primary culture (Dulbecco’s Modified Eagle Medium (DMEM): Nutrient Mixture F-12) were bought from Gibco BRL Life Technologies (Rockville, MD, USA). Hepatocyte Culture Medium and Hepatocyte High Performance Medium were from Upcyte Technologies (Hamburg, Germany). TRI reagent was purchased from the Molecular Research Center (Cincinnati, OH, USA). TaqMan systems for the analysis of *ABCB1, ABCG2* and *ABCB1* mRNA expression, gb Reverse Transcription Kit and gb Easy PCR Master Mix were purchased from Generi Biotech (Hradec Kralove, Czech Republic). The P450-Glo CYP3A4 Assay, the Screening System with Luciferin-IPA, the CellTiter-Glo Luminescent Cell Viability Assay kit, Caspase-Glo (for caspases 3/7, 8 and 9) and the CellTiter-Glo 2.0 Cell Viability Assay kit were bought from Promega (Madison, WI, USA). Cell Lysis Buffer 1067-400, BioVision (Milpitas, CA, USA). Collagen I was bought from Corning (Corning, NY, USA). Mouse monoclonal anti-cytokeratin 18 antibody [C-04] (FITC) (cat. no. ab52459) and anti-β-actin (cat. no. ab8226) were purchased from Abcam (Cambridge, MA, USA). Anti-ABCB1 (cat. no. sc-13131), anti-ABCG2 (cat. no. sc-377176), anti-ABCC1 (cat. no. sc-18835) and m-IgG kappa BP-HRP (cat. no. sc-516102) were bought from Santa Cruz Biotechnology (Dallas, TX, USA). All other chemicals and reagents were of the highest purity commercially available.

### 4.2. Cell Lines and Primary-Like Proliferating Cell Cultures

In this study, we used the parental variants of Madin-Darby canine kidney II (MDCKII), HL60 and A431 cells together with their counterparts overexpressing ABCB1, ABCG2 or ABCC1 transporters. Furthermore, HepG2-CYP3A4, Caco-2, LS174T, HepaFH3, NCI-H1299 and A549 cell lines were employed. The cells were obtained and cultivated as mentioned in our recently published papers [5,9,18,19,20]. The amount of DMSO (solvent for tepotinib and some of the model compounds) did not exceed 0.5% in cellular experiments. Possible distortions of results due to the use of this solvent were eliminated by using vehicle controls.

### 4.3. Preparation of Primary NSCLC Explants from Patients’ Tumor Biopsies

Tumor biopsies were donated by NSCLC patients at the Department of Cardiac Surgery, University Hospital Hradec Králové following written informed consent approved by the University Hospital Ethics Committee (study no. 202002 S04P). Subjects’ characteristics are shown in Supplementary Materials (Table S1). The NSCLC samples were collected immediately after the lung lobectomy and excision of tumor by the pathologist, which was followed by the isolation of cancer cells using a modified procedure based on previously published protocols [21]. The tumor tissues were minced in small pieces around 2–5 mm in diameter using a scalpel. To liberate the cells from tissues, the minced pieces were transferred into the prewarmed 0.1% collagenase in 1 × MEM and placed in the water bath for 30 min. Following the incubation, BSA in MEM was added and cells were sieved through a 40 μm-sized cell strainer. Then, the solution was centrifuged at 200× *g* for 5 min. The pellet was resuspended with a BSA solution in MEM. In order to remove cell debris, erythrocytes and tissue fragments, the mixture was centrifuged with Ficoll Paque Plus at 100× *g* for 10 min. Afterwards, the cells were collected from the interface and transferred into c-based media and the mixture was centrifuged for 5 min at 200× *g* to eliminate the Ficoll solution. Finally, the pellet was resuspended with c-based media and seeded in a collagen I-coated flask. The medium was replaced every second or third day. Once the confluence raised to 60–70%, fibroblasts were removed from the primary NSCLC culture using anti-fibroblast microbeads (Miltenyi Biotec, Bergisch Gladbach, Germany) according to manufacturer’s protocol. After fibroblast removal, the culture was incubated again in c-based media. Upon reaching 70% confluence, the media was replaced with media containing 10% FBS to eliminate possible traces of physiological cells. As a final step, staining of cells with anti-cytokeratin 18 antibody was used to confirm the epithelial origin of cells using flow cytometric analysis (Sony SA3800 spectral cell analyzer, Sony Biotechnology, San Jose, CA, USA). Representative dot-plot is shown in Supplementary Materials (Figure S1). Primary cells were used for experiments only up to 4 passages.

### 4.4. Inhibitory Accumulation Assays for ABC Efflux Transporters

Accumulation assays were performed as described previously [9,18,19,20]. Accumulation assays with fluorescence probe substrates hoechst 33342 and calcein AM, were performed in MDCKII-par, MDCKII-ABCB1, MDCKII-ABCG2 and MDCKII-ABCC1 (seeding densities of 5.0 × 10^4^, 5.0 × 10^4^, 5.5 × 10^4^ and 6.0 × 10^4^ cells/well, respectively) on 96-well plate. Following 24 h incubation at standard conditions, media was replaced with several concentrations of tepotinib in Opti-MEM or specific inhibitors: LY335979 (1 µM) for ABCB1, Ko143 (2 µM) for ABCG2 and MK571 (50 µM) for ABCC1. Afterwards, 8 µM hoechst 33342 or 2 µM calcein AM was added and fluorescence was monitored in bottom mode using excitation/emission wavelengths of 350/465 and 485/535 nm, respectively, using a microplate reader (Infinite M200 Pro, Tecan, Männedorf, Switzerland). The daunorubicin and mitoxantrone accumulation assays were performed in above mentioned cell lines and were also adopted to primary NSCLC cultures. Cells were seeded on 12-well plates with seeding densities of 22.0 × 10^4^, 15.0 × 10^4^, 25.0 × 10^4^ and 22.0 × 10^4^ cells/well, for MDCKII-par, MDCKII-ABCB1, MDCKII-ABCG2 and MDCKII-ABCC1 cell lines, respectively, and of 15.0 × 10^4^ cells/well for primary explants. Cells were incubated up to 70–80% confluence for approximately 24 h. After incubation, cells were washed and tepotinib dilutions in Opti-MEM or previous mentioned model inhibitors were added and incubated for 10 min. Afterwards, fluorescent drugs (2 µM daunorubicin or 5 µM mitoxantrone) were added to the cells and incubated for 1 h. The cells there trypsinized on ice, resuspended with 2% FBS in cold PBS solution and then fluorescence was detected using flow cytometer (BD FACSCanto II, Allschwil, Switzerland). The excitation/emission wavelengths for daunorubicin and mitoxantrone were 490/565 and 640/670 nm, respectively. For data presentation, the ratios of relative fluorescence units (RFUs) from treated samples to RFUs from vehicle-treated (control) cells were computed generating “relative substrate accumulation”.

### 4.5. Inhibitory Assay for Human Recombinant CYP Isoforms

CYP inhibitory assay was performed using Vivid CYP450 screening kit as described previously [9,18,19,20]. The kits consist of microsomal fraction containing human CYP isoforms, human CYP reductase and in several cases cytochrome b5. Experiments were performed following the manufacturer’s protocol on black 96-well plates using kinetic mode. Dilutions of tepotinib or model inhibitors were prepared in buffer and platted in the wells. Then, the master mix (CYP isoenzyme with an NADPH regeneration system in buffer) was added in each well and plate was pre-incubated for 10 min. The reaction was started with the mixture of NADP^+^ and Vivid substrate and the fluorescence of samples was measured in 1 min interval for 60 min using an Infinite M200 Pro microplate reader (Tecan, Männedorf, Switzerland). Data from linear phase (15 min) were used for data evaluation.

### 4.6. Inhibition of CYP3A4 in Intact HepG2-CYP3A4 Cells

This method was performed using the P450-Glo CYP3A4 Assay and Screening System with Luciferin-IPA together with the CellTiter-Glo Luminescent Cell Viability Assay as described previously [9,19,20]. HepG2-CYP3A4 cells were seeded at the density of 8.0 × 10^4^ cells/well on a 96-well plate and incubated for 24 h. Then, the cells were washed with 1 × PBS and tepotinib and ketoconazole dilutions in Opti-MEM were added to them. Plates were pre-incubated for 10 min at standard conditions and then, luminogenic CYP3A4 substrate luciferin-IPA was added to each well with the except for background. Plates were then incubated for 45 min at room temperature. Afterwards, cells were placed on ice and the culture media from the wells were transferred to an opaque white 96-well plate. Luciferin Detection Reagent was added in a ratio of 1:1 (*v*/*v*) and the plate was incubated for 20 min at room temperature. The luminescence was measured using a microplate reader (Infinite M200 Pro, Tecan) with an integration time of 250 ms. Concurrently, to the cells were incubated for 30 min together with 35 µL of pure Opti-MEM. After incubation, 25 µL of CellTiter-Glo Reagent was added and cells were shaken for 2 min. The lysates were incubated at room temperature for 10 min and then, they were transferred to an opaque white 96-well plate and luminescence was measured at the conditions used for CYP3A4 activity assessment. Relative luminescence units (RLUs) from luciferin-IPA metabolite were normalized to viability RLUs obtained with CellTiter-Glo kit.

### 4.7. Proliferation MTT and XTT Assays

These colorimetric assays were used in various studies presented in this paper (prior induction studies, MDR reversal experiments and comparative viability assays) and were performed as reported previously [9,18,19,20]. MTT was used in primary NSCLC cultures and in all cell lines with the except for suspension HL60 cells, for which XTT assay was used. In MTT experiments, cells were seeded on a 96-well culture plate at densities (in number of cells/well) of 1.3 × 10^4^ for MDCKII-par, MDCKII-ABCB1 and MDCKII-ABCG2; 1.2 × 10^4^ for A431-par, A431-ABCB1, A431-ABCG2 and A431-ABCC1; 1.2 × 10^4^ for NCI-H1299; 1.0 × 10^4^ for A459; 2.0 × 10^4^ for Caco-2; 5.0 × 10^4^ for LS174T; and 1.0 × 10^4^ for primary NSCLC explants. On the other hand, for XTT cells were seeded with densities 2.0 × 10^4^ for HL60-par, HL60-ABCB1, HL60-ABCG2 and HL60-ABCC1 cells/well on 96-well plates. For MTT assay, cells were seeded and cultured for 24 h before drug addition, whereas for XTT, drugs were added immediately after seeding. Following 48 h incubation with drugs, Opti-MEM solutions of MTT or XTT (1 mg/mL) with phenazine methosulfate solution (7.50 μg/mL; only for XTT) was added and the cells were incubated for 1 h with the exception of HL60s that were incubated for 5 h. In MTT experiments, cells were lysed with DMSO, in XTT ones, the level of formazan was measured directly. Absorbance measurements for MTT (570 nm, 690 nm as background) or XTT (450 nm) were performed with a microplate reader (Infinite M200 Pro, Tecan).

### 4.8. Drug Combinations

This assay was performed in the similar manner as described previously [9,18,19,20]. Experiments with MDCKII, HL60, A431 cell lines and NSCLC patient-derived explants followed the methodology described in the subsection above. Drug combination effects were quantified using combination index (CI) method of Chou-Talalay with CompuSyn 3.0.1 software (ComboSyn Inc., Paramus, NJ, USA). Based on the computed CI values, the effects of drug combinations were classified as synergistic (CI < 0.9), additive (0.9 < CI < 1.1) or antagonistic (CI > 1.1) [22].

### 4.9. MDCKII Cellular Monolayer Transport Assay and UHPLC-MS/MS Analysis

This assay was performed as described previously [9]. For the formation of the monolayer, MDCKII cells were seeded at a density of 1.5 × 10^6^ cells per insert. The Transwells were incubated until full confluence for 7 days with media replacement every 2 days. Prior addition of tested drugs, inserts were washed with 1 × PBS on both apical and basal sides. At the beginning of the experiments, Opti-MEM with or without the model inhibitor (1 µM LY335979) was added and cells were preincubated for 10 min. Afterwards, the medium was replaced with 1 µM tepotinib in Opti-MEM (with or without model inhibitor) in the donor chamber. Samples were then collected from the acceptor chambers at 0.5, 1, 2 and 4 h intervals. Cellular monolayers’ integrity was verified using fluorescein isothiocyanate-labeled dextran (MW = 40 kDa) accepting leakage up to 5% per hour. Detection of tepotinib was performed on the Agilent 1290 Infinity II UHPLC system coupled to the Agilent 6470 QqQ mass spectrometer similar as in previous study [9]. MS source parameters were set for tepotinib as follows: drying gas 320 °C at 10 L/min, sheath gas 400 °C at 12 L/min, nebulizer pressure 30 psi, capillary voltage 3000 V and nozzle voltage 0 V. Transitions of [M ^+^ H]^+^ ions *m*/*z* were detected with the setting of dwell time 150 ms, cell acceleration 4 V, fragmentor 152 V for transitions 493→112, 70 and 44 (collision energy-CE 28, 60 and 60 V).

### 4.10. Gene Expression Studies

Gene expression studies were performed employing quantitative real-time reverse transcription PCR (qRT-PCR) technique as described in our previous papers [9,18,19,20].

The Caco-2, LS174T, A549, NCI-H1299 cells or primary lung cancer cells were seeded on 12-well plates with the densities of 50 × 10^4^, 100 × 10^4^, 24 × 10^4^, 18 × 10^4^ or 15 × 10^4^ cells/well, respectively, and incubated for 24 h. After incubation, the medium was replaced with 1.5 µM tepotinib or fresh medium containing 0.15% DMSO (vehicle control). Samples were collected at 24 h and 48 h intervals after drug treatment using TRI Reagent and then, mRNA isolation was performed. Agarose gel electrophoresis was used for the control of RNA quality and integrity and the RNA yield was monitored via NanoDrop ND-1000 spectrophotometer (American Laboratory Trading, East Lyme, CT, USA). RNA (1000 ng) was transcribed to cDNA using gb Reverse Transcription Kit. *ABCB1*, *ABCG2* and *ABCC1* mRNA expressions were detected with Master Mix and TaqMan-based qRT-PCR systems using QuantStudio 6 (Life Technologies, Carlsbad, CA, USA). The geometric mean of *B2M* and *HPRT1* housekeeping genes’ levels were used as comparators for the expression of each target gene (using 2^−ΔΔCt^ method).

In the case of the HepaFH3 hepatocytes, first, viability was analyzed with CellTiter-Glo 2.0 Viability Assay; the luminescence was measured using a microplate reader (FLUOstar Omega, BMG Labtech, Ortenberg, Germany). For gene expression studies, the cells were seeded in density of 1.6 × 10^5^ cells/well on collagen I-coated 12-well plates and incubated to full confluence for 4 days. After incubation, medium was exchanged with 1.5 µM tepotinib or vehicle control (0.1% DMSO). RNA was isolated using InnuPREP RNA Mini Kit from Analytik Jena (Jena, Germany) at 72 h. RNA integrity and quality was checked by agarose gel electrophoresis and the concentration assessed by NanoDrop. Revert aid reverse transcriptase and 1 µg of DNase digested RNA was used for the reverse transcription. Primers for qRT-PCR target genes (*CY1A2*, *CYP3A4* and *CYP2B6*) and housekeeping genes (*GAPDH*, *SDHA*) are presented in [2]. mRNA levels were determined using Maxima Probe qPCR Master Mix and EvaGreen in 96-well plates. Relative quantification of the examined *CYPs* was performed using the 2^−ΔΔCt^ method; the geometric mean of *GAPDH* and *SDHA* levels was used as an internal control to normalize the variability in expression levels. qRT-PCR was performed on CFX96 Touch Real-Time PCR Detection System (Bio-Rad, Hercules, CA, USA).

### 4.11. Caspase Activity Assays

This method was performed based on previously described protocol [5]. Caspase-Glo kits were used to detect the activities of caspases 3/7, 8 and 9. A431 cell sublines were seeded at densities of 3.5 × 10^4^ cells/well on 96-well culture plates and incubated for 24 h. After incubation, the medium was replaced with medium containing tested drugs and samples were subsequently collected at 6 or 24 h. Plates were transferred on ice, the medium was removed, and pre-cooled BioVision cell lysis buffer was added. Cells were lysed for 15 min on ice, and then samples were collected and stored on ice. Lysates were centrifuged at 12,000× *g* for 5 min to remove debris. Subsequently, cell lysates were transferred on a white 384-well plate and mixed with caspase activity detection reagents in a 1:1 (*v*/*v*) ratio. The plates were incubated for 1 h at room temperature. Luminescence was measured by a multiplate reader (Infinite M200 PRO, Tecan) using 250 ms integration time. The protein content of cell lysates was assessed using the Pierce BCA Protein Assay Kit; these data were used for the normalization of luminescence data.

### 4.12. Western Blotting

This method was performed with minor modifications as described previously [23,24]. Primary culture cells were seeded in Petri dishes to full confluence. Once the confluence reached 100%, the cells were washed twice with cold 1 × PBS and lysed with cell lysis buffer (20 mM Tris, 150 mM NaCl, 12.8 mM EDTA, 1 mM EGTA, 4.2 mM Na-pyrophosphate, 1 mM Na3VO4 and 10 mL/L Triton; 10 µL/mL protease inhibitor cocktail were added into cell lysis buffer before use). Whole cell lysates were centrifuged at 4 °C in 12,000× *g* for 30 min. The total protein concentration was determined by using Bradford Assay Reagent. A total of 20 μg protein from each sample was loaded in and separated by 8% SDS-PAGE. Subsequently, separated protein samples were transferred to PVDF membranes by using Trans-Blot TurboTM Transfer System (Bio-Rad Laboratories, Hercules, CA, USA). The membranes were blocked with TBST buffer (0.1% Tween-20 in TBS) containing 5% non-fat dry milk for 1.5 h at 25 °C. Then, the membranes were incubated with specific primary antibodies for 16 h at 4 °C. The monoclonal primary antibodies were diluted with TBST buffer as follows: anti-ABCB1 (1:500), anti-ABCG2 (1:1000), anti-ABCC1 (1:500) and anti-β-actin (1:10,000). After being washed three times with TBST buffer, the membranes were incubated with horseradish peroxidase-conjugated secondary antibodies (diluted in TBST buffer; 1:2000) at room temperature for 1 h. Immobilon Western Chemiluminescent HRP Substrate (EMD Millipore, Billerica, MA, USA) was applied onto the membrane, which was then scanned using Chemi DocTM MP Imaging System (Bio-Rad Laboratories). β-actin served as the internal control. Bands´ densities were analyzed by ImageJ software (version 1.46r; National Institutes of Health, Bethesda, MD, USA).

### 4.13. Molecular Docking Simulations

Tepotinib was downloaded from ZincDatabase (http://zinc.docking.org; accessed on 12 March 2019) [25], its energy was minimized using UCSF Chimera 1.14 [26] and prepared for docking with MGL Tools 1.5.6. [27]. The crystal structures of ABCB1 (PDB IDs: 6QEX and 6C0V) and ABCG2 (PDB IDs: 6HIJ and 6HBU) were obtained from the RSCB Protein Data Bank (http://www.rcsb.org; accessed on 12 March 2019, 28 March 2019, 3 May 2019 and 29 October 2019 for 6HIJ, 6HBU, 6C0V and 6QEX, respectively) [28,29,30,31,32] and prepared for docking as described previously [9,20]. Rigid and flexible docking was performed with AutoDock Vina 1.1.2 [33] into 6C0V, 6HIJ, 6HBU and 6QEX with conditions set previously [9,20]. Based on rigid docking results, the coordinates for Hoechst 33342 binding site (H-site) were modified to x = 165.44, y = 153.48, z = 186.16 (flexible residues: Arg-148, Asn-183, Glu-184, Gln-882, Asp-886, Asn-930, Lys-934, Phe-938). The coordinates for rhodamine 123 binding site (R-site) were x = 174.51, y = 173.49, z = 166.69 (flexible residues: Asn-296, Phe-303, Tyr-307, Gln-725, Phe-770, Gln-838, Asn-842, Gln-990, Val-991) and x = 174.84, y = 178.69, z = 177.66 (flexible residues: Lys-291, Asn-296, Phe-770, Gln-773, Glu-782, Lys-826, Gln-838, Phe-994). The coordinates for modulator site (M-site) were x = 173.33, y = 166.74, z = 161.48 (flexible residues Phe-303, Tyr-307, Phe-336, Ile-340, Phe-343, Gln-347, Gln-725, Phe-728, Phe-732, Phe-983, Gln-990). From our tested substrates, Hoechst 33342 binds preferentially to H-site, whereas daunorubicin to R-site and less potently also to M-site. The exhaustiveness parameter was 8 and the size of the grid box was 35 × 35 × 35. PyMOL (The PyMOL Molecular Graphics System, Schrödinger, LLC) and BIOVIA Discovery Studio Visualiser by Dassault Systèmes (San Diego, CA, USA) were used to evaluate the results.

### 4.14. Statistical Analysis

Analysis of obtained data was performed by GraphPad Prism software version 8.0.1 (GraphPad Software Inc., La Jolla, CA, USA) using one-way *ANOVA* followed by Dunnett’s post hoc test (Figure 1, Figure 5 and Figure 6B) or two-tailed unpaired *t*-test (Figure 5 and Figure 7A,B and Supplementary Materials, Table S2). Treated variants were compared with control values, if not specified otherwise. Values with *p* < 0.05 were considered statistically significant. * *p* < 0.05, ^#^ *p* < 0.05; ** *p* < 0.01, ^##^ *p* < 0.01; *** *p* < 0.001, ^###^ *p* < 0.001; **** *p* < 0.0001, ^####^ *p* < 0.0001. All results come from at least three independent repetitions, each done in biological triplicates and are expressed as the mean ± SD.

## 5. Conclusions

In conclusion, we demonstrate that tepotinib has a clear potential to become a perpetrator of pharmacokinetic DIs on ABCB1 and CYP2C9. In addition, the drug might be potentially utilized as a valuable chemosensitizer in patients suffering from NSCLC tumors expressing ABCB1 and/or ABCG2. We believe that our in vitro and ex vivo findings might serve as a valuable background for subsequent in vivo investigations, which will verify the clinical impact of suggested therapeutical approach.

## Data Availability

The authors declare that the data generated and analyzed during this study are included in this published article and associated Supplementary Materials. In addition, datasets generated and/or analyzed during the current study are available from the corresponding author on reasonable request.

## Supplementary Materials

The following are available online at https://www.mdpi.com/article/10.3390/ijms222111936/s1.
