# Supplementary material for: Tepotinib Inhibits Several Drug Efflux Transporters and Biotransformation Enzymes: The Role in Drug-Drug Interactions and Targeting Cytostatic Resistance In Vitro and Ex Vivo
Source: Int J Mol Sci. 2021 Nov 3;22(21):11936. doi: 10.3390/ijms222111936 (PMC8584989; doi:10.3390/ijms222111936)
Supplement: Supplementary file 1 [file ijms-22-11936-s001.zip › ijms-1413281-supplementary.pdf]

Supplementary Tables

Table S1. Basic characteristics of tumor biopsies donated by NSCLC patients. None of the patients underwent neoadjuvant chemotherapy.

Sample No.	gender	age	histopathological diagnosis
1	male	70	squamous carcinoma
2	male	70	squamous carcinoma
3	female	56	large cell neuroendocrine carcinoma
4	male	75	squamous carcinoma
5	male	51	large cell neuroendocrine carcinoma
6	male	73	adenocarcinoma

Table S2. IC_{50}-shift analysis of the combination effects of 5 or 10 µM tepotinib for ABCB1 or ABCG2, respectively, with substrate cytostatics in MDCKII, A431 and HL60 cell lines.

Cell line	Drug(s)	$IC_{50}{}^{a}$ (µM)	95 % CI (µM)	$R_R{}^{b}$
MDCKII-parent				
	daunorubicin	0.947	(0.814 - 1.07)	
	mitoxantrone	1.59	(1.39 - 1.64)	
	daunorubicin + tepotinib	1.00 ^{ns}	(0.894 – 1.08)	0.947
	mitoxantrone + tepotinib	1.31 ^{ns}	(1.17 – 1.47)	1.21
MDCKII-ABCB1				
	daunorubicin	11.9	(10.7 - 12.9)	
	daunorubicin + tepotinib	0.819***	(0.791 – 0.867)	14.5
MDCKII-ABCG2				
	mitoxantrone	9.98	(8.91 - 11.5)	
	mitoxantrone + tepotinib	1.47**	(1.36 - 1.69)	6.79
A431-parent				
	daunorubicin	1.99	(1.54 – 2.37)	
	mitoxantrone	2.10	(1.54 – 2.37)	
	daunorubicin + tepotinib	1.67 ^{ns}	(1.15 – 2.23)	1.19
	mitoxantrone + tepotinib	1.40 ^{ns}	(0.959 – 1.64)	1.50
A431-ABCB1				
	daunorubicin	21.7	(1.61 - 2.53)	
	daunorubicin + tepotinib	5.70****	(4.43 – 6.79)	3.80
A431-ABCG2				
	mitoxantrone	56.5	(37.86 – 93.6)	
	mitoxantrone + tepotinib	5.57***	(3.67 – 7.15)	10.1
HL60-parent				

	daunorubicin	0.0531	(0.0473 - 0.0589)	
	mitoxantrone	0.0305	(0.230 - 0.0370)	
	daunorubicin + tepotinib	0.0537 [ns]	(0.0423 - 0.0565)	0.989
	mitoxantrone + tepotinib	0.0363[ns]	(0.00301 - 0.0429)	0.840
HL60-ABCB1				
	daunorubicin	2.14	(1.61 - 2.53)	
	daunorubicin + tepotinib	0.0729****	(0.0519 - 0.111)	29.4
HL60-ABCG2				
	mitoxantrone	0.669	(0.520 - 0.823)	
	mitoxantrone + tepotinib	0.209*	(0.142 - 0.270)	3.20

[a] These values were calculated using the data shown in Fig. 3A. The IC_{50} values from the combinations were compared to those from daunorubicin or mitoxantrone alone in the appropriate cell lines using the two-tailed unpaired *t*-test (*$p < 0.05$; **$p < 0.01$; ***$p < 0.001$; ****$p < 0.0001$).

[b] Reversal ratio (R_R) is the ratio of the IC_{50} from single drug treatment to that from the combined drug treatment in the cell line of interest.

Supplementary Figures

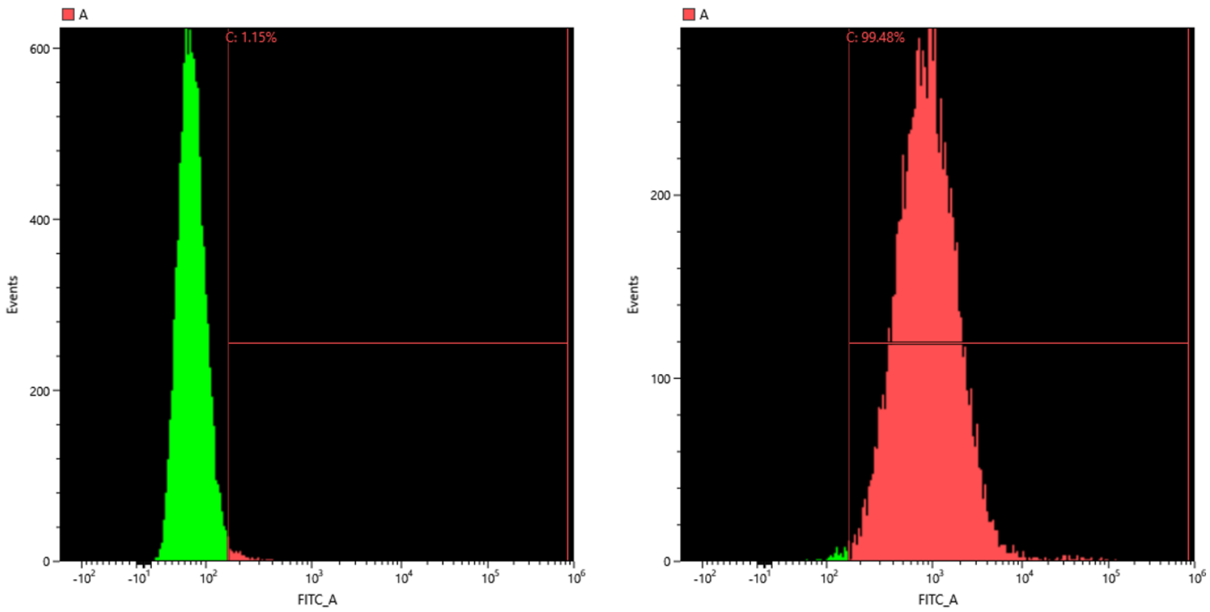

Figure S1. Representative histograms from the analysis of origin of isolated primary NSCLC cells. The cells isolated from lung tumors were characterized as epithelial via the expression of cytokeratin 18 using flow cytometry. Histograms represent isotype control (green-filled) versus cytokeratin 18-positive cells (pink-filled).

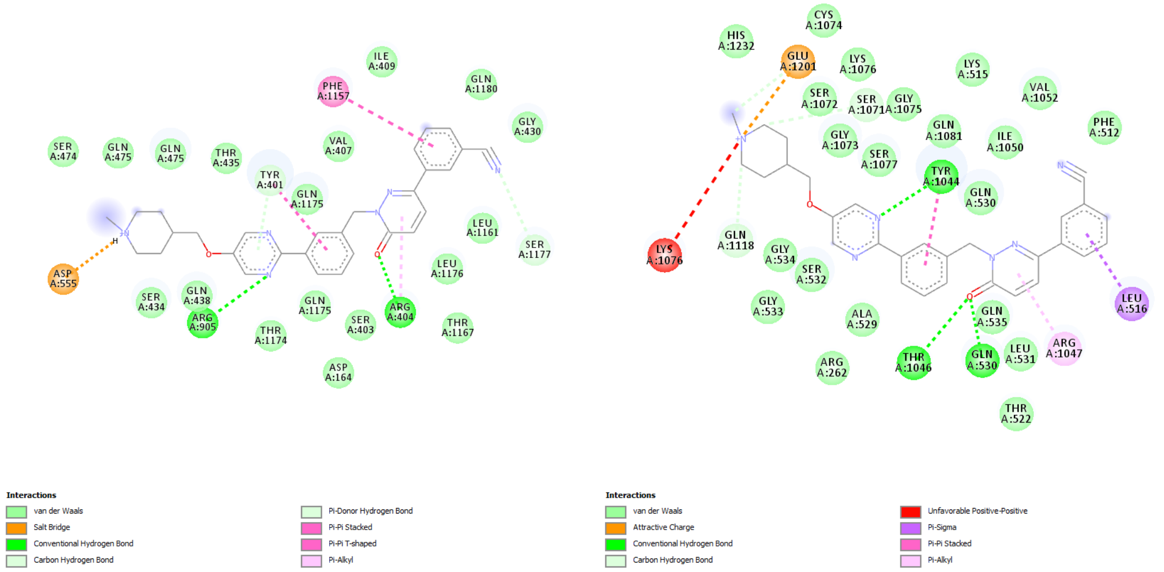

Figure S2. Two-dimensional diagrams of predicted interactions of tepotinib with nucleotide binding domain 1 (left; -11.3 kcal/mol) and nucleotide binding domain 2 (right; -11.9 kcal/mol) of ABCB1.
